# Diabetes and depression in Africa: Causality, relationship and clinical implications

**DOI:** 10.1016/j.amsu.2022.104422

**Published:** 2022-08-17

**Authors:** Damilola Quazeem Olaoye, Olayemi Amusile, Sejoro Sehubo Tonuewa, Oluwapelumi Andrew Eseola, Temitope Ben Ajepe, Omobolanle Fausat Adekoya

**Affiliations:** Faculty of Pharmacy, University of Ibadan, Nigeria; Lakeshore Cancer Centre, Victoria Island Lagos, Nigeria; Federal Medical Centre Asaba, Delta State, Nigeria; Faculty of Pharmacy, University of Lagos, Nigeria; Faculty of Pharmacy, University of Ibadan, Nigeria; Dora Akunyili College of Pharmacy, Igbinedion University, Okada, Edo State, Nigeria; University College Hospital, Ibadan, Nigeria

## Abstract

The public health threats posed by diabetes and depression individually have been well emphasised globally. However, it is increasingly important to understand these diseases' causes, relationships, and implications in comorbid states. Depression, anxiety, and diabetes are the top 10 causes of disability-adjusted life years (DALYs) in countries worldwide. Several reports have also suggested depression to occur two to three times more frequently in people with diabetes mellitus; with the majority of the cases remaining under-diagnosed, the causes and relationship remain rather complex and understudied. Although the exact link between depression and diabetes is yet to be detailed, quite a number of studies have supported that there is a bidirectional relationship between diabetes and depression. As Pieces of evidence, theories and reports continue to shed more light on the cause and relationship between these two conditions, its implications remain understudied, especially in low-middle-income settings. This commentary draws out the need for intentional screening for depressive symptoms in diabetic patients as early as possible from the implications of its neglect. It is, however, recommended that in the management of diabetes mellitus, emphasis should also be placed on depression as a co-morbidity, just as certain other disease conditions like hypertension and dyslipidaemia are considered.

## 1 Depression and diabetes

The public health threat posed by diabetes and depression individually have been well emphasised globally. However, it is increasingly important to understand the causes, relationship and implications of these diseases in comorbid states.

Diabetes mellitus (DM) is a well-studied metabolic disorder of carbohydrate, protein, and fat metabolism often characterized by chronic hyperglycaemia [1]. While Depression is a common mental disorder characterised by symptoms ranging from fatigue, lack of concentration, insomnia to suicidal thoughts. The variety of presenting symptoms associated with depression are said to have a considerable effect on patients’ health-related quality of life and satisfaction with medical care.

The International Diabetes Federation reports that “among individuals between the ages of 20 and 79 years, about 24 million are living with diabetes in 2021 and it is estimated to increase to 33 million and 55 million by 2030 and 2045 respectively. On the other hand, the World Health Organisation had earlier reported in 2017 that of the 322 million people living with depression about 29.9 million are living in Africa [2]. Both diabetes and depression are among the top contributors to the global disease burden [2]. Depression, anxiety, and diabetes are top 10 cause of disability adjusted life years (DALYs) in countries around the world. Several reports _h_ave also suggested depression to occur two to three times more frequently in people with diabetes mellitus with majority of the cases remaining underdiagnosed, the causes and relationship remain rather complex and understudied [3]. In this commentary, the relationship, causality, and implication of depression in diabetic patients in clinical practices will be elucidated.

## 2 Causalty and relationship

In a meta-analysis aimed to determine the association of depression and the risk of diabetes, it was estimated that depression increased the risk of developing diabetes mellitus and type 2 diabetes mellitus by 41% and 32% respectively [4].

In 2015, two reviews pointed out three possible reasons for the association of diabetes and depression; both diseases likely have similar aetiology; diabetes facilitates the prevalence or risk for future depression with depression also facilitating the prevalence or risk for future diabetes [3]. Researchers using a decade of data from Nurses’ Health Study identified a 17% rise in diabetes risk for individuals with depression and a 29% rise in depression risk for those with diabetes [5]. Similarly, another study shows that the predominance of depression may be up to three-times higher in patients with type 1 diabetes and twice as high in people with type 2 diabetes compared with Global population [3]. The link between depression and diabetes is robust across methods and populations, as stated by another study which claims depression among individuals living with diabetes is estimated to range from 9% to 35%. Interestingly both diabetes and depression have been listed as top causes of disability adjusted life years (DALYS), while depression and anxiety are the 4th cause, diabetes is the 8th cause of disability adjusted life years in developed countries [3].

The increased risk for diabetes associated with depression remains even when other known diabetes risk factors are considered, including poor diet, family history, inflammation, some antidepressant, and sedentary lifestyle [5]. Treatment studies suggests a casual association between diabetes and depression although level of casualty may be complicated. When depression is treated in individuals diagnosed with diabetes, haemoglobin A1C (HbA1C), a glucose control biomarker, declines moderately [5].

The fusion of depression and diabetes in low- and middle-income countries may enhance the emergence of diabetic complications and finally rise in morbidity and mortality rate [6]. As a continent, Africa has no record of a sum total estimation of depression prevalence among diabetes mellitus patients at a regional [7]. However, results presented from a systematic review [6] involving four regions in Africa estimated the prevalence rate of depression among patients with diabetes in Africa to be 40% across African regions. Compared to a global estimate the findings reported more prevalence of depression in diabetic patients in Africa in contrast to lower cases in Europe (24%), Australia (29%) and Asia (32%) [6].

## 3 Implications of depression in diabetic patients in clinical practice

It has been established through various studies that depression and diabetes both have a negative impact on the quality of life of patients living with either of them. However, when they co-exist, they impact quality of life even more negatively [8]. It is often safe to infer that the occurrence of depression and diabetes together can possibly make the conditions more difficult to manage. This difficulty in management of patients presenting with depression and diabetes increases the risk of complications such as dementia, stroke and even mortality. There are reported possibilities that depression can heighten the psychological impact of being diagnosed with diabetes and thereby result in an increased risk of diabetes related distress. A review by Badescu et al. [3], states that “depression has a synergistic effect in patients with DM1 and DM2, increasing the risk for complications of both micro- and macro-vascular nature, increased hyperglycaemia, predicting greater mortality.” Schram et al. [8], further notes that depression negatively impacts the ability of diabetic patients to take care of themselves. The study suggests that there may be a possibility of depressive symptoms being predictive of functional disability in patients with diabetes in future as several evidence suggests a causal relationship between depression and functional disability. A meta-analysis by Gonzalez et al. [9], also suggests that depression has a negative effect on the adherence of diabetic patients to self-care with the worst factor hit being missed appointments.

## 4 Conclusion and recommendation

In the management of diabetes mellitus, emphasis should also be placed on depression as a comorbidity just as certain other disease conditions like hypertension and dyslipidaemia are considered. Every diabetic patient should be screened periodically for depression and managed as such if need be [10]. In depressed diabetic patients’ priority should be given to early detection of depressive symptoms to ensure that the depressive state does not negatively impact the clinical management of diabetes.

A multidisciplinary approach is also recommended in clinical management, taking into accounts the available facts and current gaps in early identification of diabetic patients with depressive symptoms.

## Ethical approval

Not applicable.

## Sources of funding

No funding

## Author contribution

This review was conceptualized by Damilola Quazeem Olaoye and Omobolanle Fausat Adekoya. Sejoro Sehubo Tonuewa, Olayemi Amusile, Oluwapelumi Andrew Eseola, and Damilola Quazeem Olaoye contributed to writing the article. Temitope Ben Ajepe, Olayemi Amusile and Sejoro Sehubo Tonuewa further reviewed the first draft of the manuscript. All the authors read and approved the final manuscript.

## Registration of research studies

1. Name of the registry:
2. Unique Identifying number or registration ID:
3. Hyperlink to your specific registration (must be publicly accessible and will be checked):

## Guarantor

All named authors accept full responsibility to publish manuscript.

## Consent for publication

All authors agreed on the publication of this manuscript.

## Availability of data and materials

Not Applicable.

## Provenance and peer review

Not commissioned, externally peer reviewed.

## Consent

Not Applicable.

## Declaration of competing interest

We declare that there are no conflicts of interest.
